# Screening and identification of key chromatin regulator biomarkers for ankylosing spondylitis and drug prediction: evidence from bioinformatics analysis

**DOI:** 10.1186/s12891-023-06490-y

**Published:** 2023-05-16

**Authors:** Han Wang, Hongbo Jin, Zhiyang Liu, Chengju Tan, Lin Wei, Mingfen Fu, Yizhuan Huang

**Affiliations:** Acupuncture and Massage Department, Affiliated Sport Hospital of CDSU, number 251, Wu Hou Ci Da Jie, Cheng Du, Si Chuan, 610041 China

**Keywords:** Ankylosing spondylitis, Chromatin regulator, Pathogenic genes, Bioinformatics, Mechanism

## Abstract

**Background:**

Ankylosing spondylitis (AS) is one of the most common immune-mediated arthritic diseases worldwide. Despite considerable efforts to elucidate its pathogenesis, the molecular mechanisms underlying AS are still not fully understood.

**Methods:**

To identify candidate genes involved in AS progression, the researchers downloaded the microarray dataset GSE25101 from the Gene Expression Omnibus (GEO) database. They identified differentially expressed genes (DEGs) and functionally enriched them for analysis. They also constructed a protein–protein interaction network (PPI) using STRING and performed cytoHubba modular analysis, immune cell and immune function analysis, functional analysis and drug prediction.The results showed that DEGs were mainly associated with histone modifications, chromatin organisation, transcriptional coregulator activity, transcriptional co-activator activity, histone acetyltransferase complexes and protein acetyltransferase complexes.

**Results:**

The researchers analysed the differences in expression between the CONTROL and TREAT groups in terms of immunity to determine their effect on TNF-α secretion. By obtaining hub genes, they predicted two therapeutic agents, AY 11–7082 and myricetin.

**Conclusion:**

The DEGs, hub genes and predicted drugs identified in this study contribute to our understanding of the molecular mechanisms underlying the onset and progression of AS. They also provide candidate targets for the diagnosis and treatment of AS.

## Introduction

Ankylosing spondylitis (AS) is an immune-mediated spondyloarthropathy, the cause of which is not fully understood [1]. It is a debilitating arthritic condition affecting the spine, with onset typically occurring before the age of 40 and a male predominance, with a prevalence of approximately 0.5% in the USA [2]. AS is characterized by inflammatory erosive osteopenia and atypical bony overgrowth. In the spine, syndesmophyte formation between vertebrae ultimately leads to the development of the characteristic "bamboo spine" [3].

AS mainly affects the axial skeleton, especially the sacroiliac joints and the spine.The pathophysiology of AS involves enthesitis, inflammation of the sites where ligaments, tendons, and joint capsules attach to bone. This leads to bone erosion, new bone formation, and eventual fusion of the joints, resulting in stiffness, pain, and limited mobility.Late-stage AS can lead to severe spinal deformities, including hyperkyphosis and loss of lumbar lordosis, due to the progressive ankylosis of the vertebral column. In addition, calcification and ossification of the paraspinal ligaments and other soft tissues can occur, leading to further spinal fusion and permanent stiffness.The management of AS involves a multidisciplinary approach, with pharmacological therapy and non-pharmacological interventions. Nonsteroidal anti-inflammatory drugs (NSAIDs), corticosteroids, disease-modifying antirheumatic drugs (DMARDs), and biologic agents are commonly used to control pain and inflammation. Physical therapy, exercise, and postural management are also essential to improve function and prevent deformities. In severe cases, surgical intervention may be necessary to correct spinal deformities or joint damage.

Furthermore, the early signs of AS may not be readily apparent, and many patients do not receive a diagnosis at the outset, resulting in an 8–10 year lag between symptom onset and definitive diagnosis [4]. The presence of the human leukocyte antigen (HLA)-B27 gene in several populations provides further evidence that aberrant gene expression and mutation play a role in the development and progression of AS. Notably, the major histocompatibility complex (MHC) class I allele B27 (HLA-B27) is associated with 90% of patients with AS and can therefore serve as a genetic biomarker for diagnosis [5]. However, the exact mechanisms by which HLA-B27 induces disease remain unclear. Recently, variants of the M1-aminopeptidase gene ERAP1 and the adjacent related gene ERAP2 were found to be associated with AS. Both proteins participate in peptide trimming in the endoplasmic reticulum, influencing the length and amino acid composition of peptides available for HLA class I presentation [6]. Additionally, there is a strong correlation between AS and the immune system. However, due to the lack of effective diagnostic methods in the early stages of the disease, timely diagnosis and treatment for AS are challenging. Therefore, understanding the precise mechanisms of chromatin regulators involved in AS and their relevance to the immune system is crucial for developing effective diagnostic and therapeutic strategies.

Over the past few decades, microarray technology and bioinformatic analysis have been extensively utilized to screen for genetic alterations at the genome level, leading to the identification of differentially expressed genes (DEGs) and functional pathways implicated in the chromatin regulators of AS. Our research aims to investigate the involvement of chromatin regulatory factor-related genes in the pathogenesis of AS and explore drug prediction strategies, offering novel insights into the treatment of AS.

## Materials and methods

### Data download and processing

We downloaded an AS dataset from the Gene Expression Omnibus (GEO) for the analysis of possible diagnostic markers. GSE25101 [7], the dataset contains a total of 32 samples. We first matched the probe names of the datasets to their corresponding gene symbols using the programming language R (version 4.1.3) and then, de-batched and normalized the datasets so that they could be analyzed at the same level. We combine the AS samples from the dataset and controls into one file for uniform analysis. Extraction of chromatin regulators-related gene expression [8]. In this study, we used the transitive language PERL for text processing and the transitive language R (version 4.1.3) for statistical analysis and image plotting.

### Differential expression analysis and construction of heat and volcano plots

To analyze the differentially expressed genes of chromatin regulatory factors between the AS and control groups, we utilized the ‘limma’ package to conduct a comprehensive analysis of the two cohorts. The cut-off values were set to |logFC|filter = 0.2 and adjusted to *p* < 0.05. The differential expression details of genes are summarized in Table 1. We further utilized the ‘pheatmap’ package to generate heat maps of differentially expressed genes, while the ‘dplyr’ package, ‘ggplot2’ package, and ‘ggrepel’ package were used to produce volcano maps highlighting genes with significant differential expression.

**Table 1:** | List of differentially expressed genes in the GEO dataset

id	logFC	AveExpr	t	*P*. Value	adj.P.Val	B
DNMT1	-0.50477	10.23597	-4.80558	0.00003	0.00877	2.34864
SETD1A	-0.40555	8.37389	-4.63131	0.00005	0.00877	1.8702
BBX	-0.39115	10.24744	-4.60326	0.00006	0.00877	1.79344
EP300	-0.52707	9.07958	-4.56155	0.00007	0.00877	1.67949
HMGB2	0.64998	8.52865	4.53017	0.00007	0.00877	1.59387
BRPF1	-0.37754	9.93934	-4.41717	0.0001	0.01016	1.28659
BCOR	-0.21043	7.71227	-4.28371	0.00015	0.01282	0.92591
DHX30	-0.29791	8.36195	-4.23063	0.00017	0.01287	0.78322
DDB1	-0.22397	8.43014	-4.19538	0.00019	0.01287	0.68874
RAI1	-0.23433	7.672	-3.98077	0.00035	0.0214	0.11832
PARP1	-0.46126	10.14424	-3.93383	0.0004	0.02204	-0.00521
SMARCC2	-0.28885	8.27497	-3.90605	0.00044	0.02204	-0.07806
CBX1	0.265	8.05086	3.84399	0.00052	0.02424	-0.24023
ENY2	0.66792	9.61061	3.7427	0.00069	0.02557	-0.50285
EP400	-0.34281	8.96964	-3.72919	0.00072	0.02557	-0.5377
BAZ1B	-0.31586	8.48301	-3.62663	0.00095	0.03089	-0.80053
ZMYND8	-0.2436	7.70101	-3.57418	0.0011	0.0334	-0.93381
MTA2	-0.33086	8.74128	-3.51711	0.00129	0.03679	-1.07788
NCOA5	-0.23606	8.95394	-3.47749	0.00143	0.03786	-1.1773
TRIM28	-0.36817	8.90798	-3.46114	0.0015	0.03793	-1.21821
EID2	-0.22543	7.33718	-3.41741	0.00169	0.03959	-1.32714
SMARCD3	0.36278	7.9723	3.40664	0.00174	0.03959	-1.35389
RIT1	0.33366	8.11547	3.40195	0.00176	0.03959	-1.36551
HCFC1	-0.46464	9.74747	-3.35499	0.002	0.04334	-1.48153
FTO	-0.2604	7.52449	-3.34066	0.00207	0.04349	-1.51679
ZBTB4	-0.34922	9.6394	-3.27133	0.0025	0.04808	-1.68636
GATAD2B	-0.42864	9.24592	-3.25801	0.00259	0.04808	-1.71873
TAF6	-0.20846	7.32041	-3.25049	0.00264	0.04808	-1.73697
ATN1	-0.22459	7.04417	-3.22981	0.00279	0.04808	-1.78706

### GO enrichment analyses of DEGs

To gain further insight into the molecular functions (MF), biological processes (BP), cellular composition (CC), and pathway enrichment of the differentially expressed genes, we conducted GO enrichment analysis using the “clusterProfiler”, “enrichplot”, and “GOplot” packages. Specifically, the “enrichplot” and “GOplot” packages were employed for GO enrichment and pathway analysis of differentially expressed genes. The cut-off values were set and adjusted to *p* < 0.05, and the results were visualized using bubble plots.

### PPI network construction/hub genes

We used the Search Tool for the Retrieval of Interacting Genes to predict the PPI network. Analyzing the functional interactions between proteins can offer insights into the mechanisms involved in the generation or development of diseases. For this study, we constructed PPI networks using the STRING database (version 11.5) [9], and interactions with combination scores > 0.4 were considered statistically significant. Cytoscape (version 3.9.1) is an open-source bioinformatics software platform that can visualize molecular interaction networks [10]. We mapped the PPI networks using Cytoscape and analyzed them locally with the Cytoscape plugin "cytoHubba" (version 4.4.6). The top 10 expressed genes were selected as hub genes.

### Immune cell and immune function analysis

We combined the AS genes obtained with genes related to immune cells and immune function obtained from ESTIMATE (https://bioinformatics.mdanderson.org/estimate/index.html) using the language R (version 4.1.3) using the "limma" package, "ggpubr "package", "reshape2" package, "GSVA" package, "GSEABase" package The "pheatmap" package, "ggpubr" package, "reshape2" package, "GSVA" package, "GSEABase" package and "pheatmap" package for statistical analysis and image plotting. We utilized the "psych" package and the "ggcorrplot" package to analyze differential gene expression in immune cells and immune function, as well as the correlation of hub genes with immune cells and immune function.

### Drug forecasting

To treat AS, a chronic immune-related disease, and to identify potential drugs that can improve the condition, we conducted a comprehensive analysis of genes that are closely related to AD. We used the hub genes to predict potential drugs via Enrichr (https://maayanlab.cloud/Enrichr/) using the DSigDB database.

## Results

### Differential expression analysis, construction of heat map, and volcano map

**Fig. 1:**
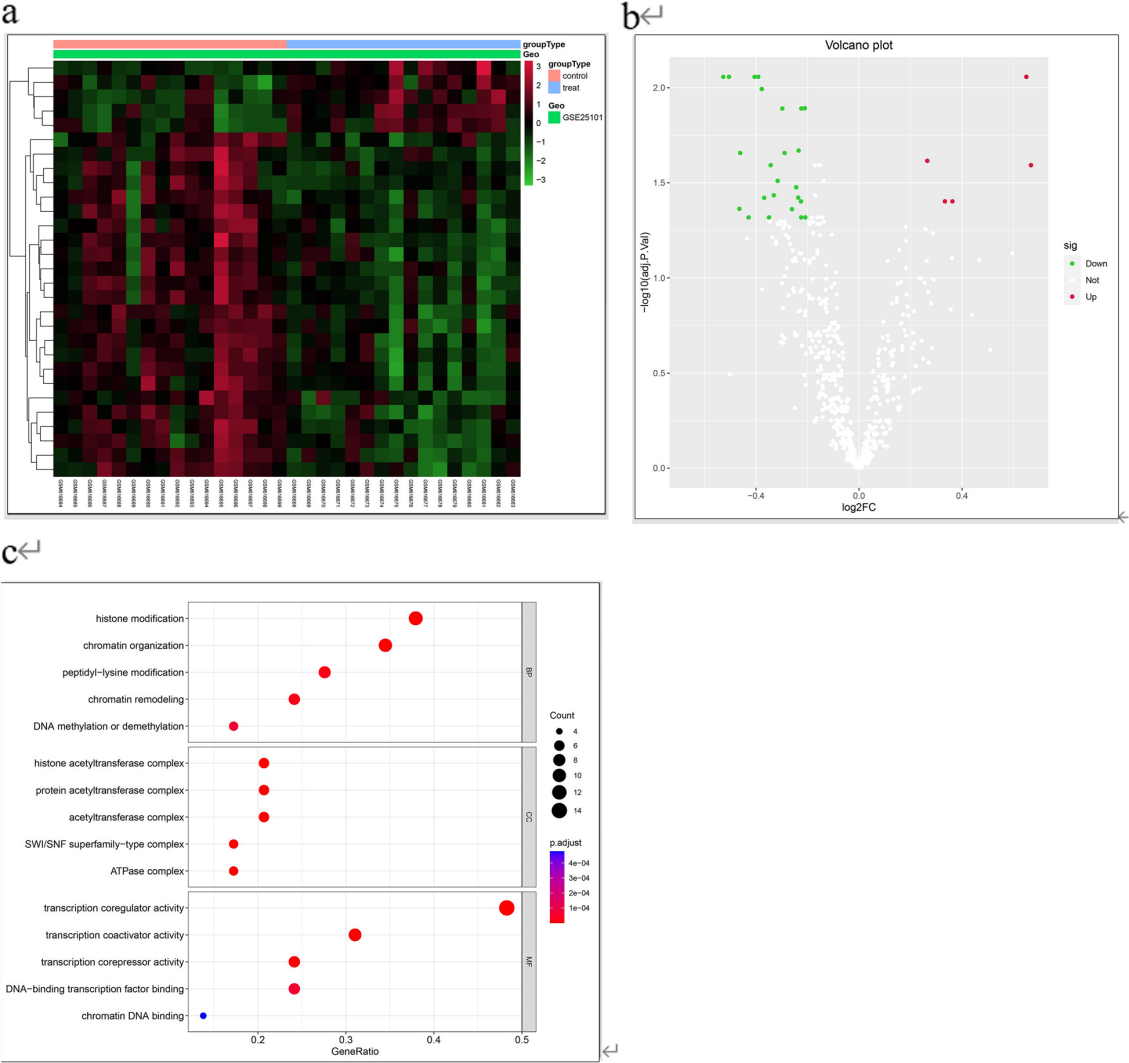
**a** Heat map of 29 differentially expressed genes associated with chromatin regulators (**b**) Volcano plot of up-and down-regulated genes in differentially expressed genes (**c**) Bubble plots are used to show the locations of DEGs enriched in BP, MF, and CC in GO analysis

We downloaded the GSE25101 dataset from the GEO database, which contained 18 AS samples and 18 control samples. After normalizing the datasets and removing inter-batch differences, we extracted gene expression data related to chromatin regulators for differential expression analysis. In total, 29 differentially expressed genes associated with chromatin regulators were identified and visualized in a heat map (Fig. 1a). Additionally, we generated a volcano plot to visualize the differential expression of these genes (Fig. 1b), revealing 4 highly expressed genes and 24 lowly expressed genes that were significantly different.

### Go enrichment analyses of degs, construction of bubble chart

GO analysis revealed that differentially expressed genes (DEGs) were significantly enriched in biological processes (BP) related to histone modification and chromatin organization. In terms of molecular function (MF), DEGs were primarily enriched in transcription coregulator and coactivator activities. With respect to cellular components (CC), DEGs were mainly enriched in the histone acetyltransferase and protein acetyltransferase complexes. These results are shown in the bubble chart in Fig. 1c.

### PPI network construction, hub gene selection, and analysis

**Fig. 2:**
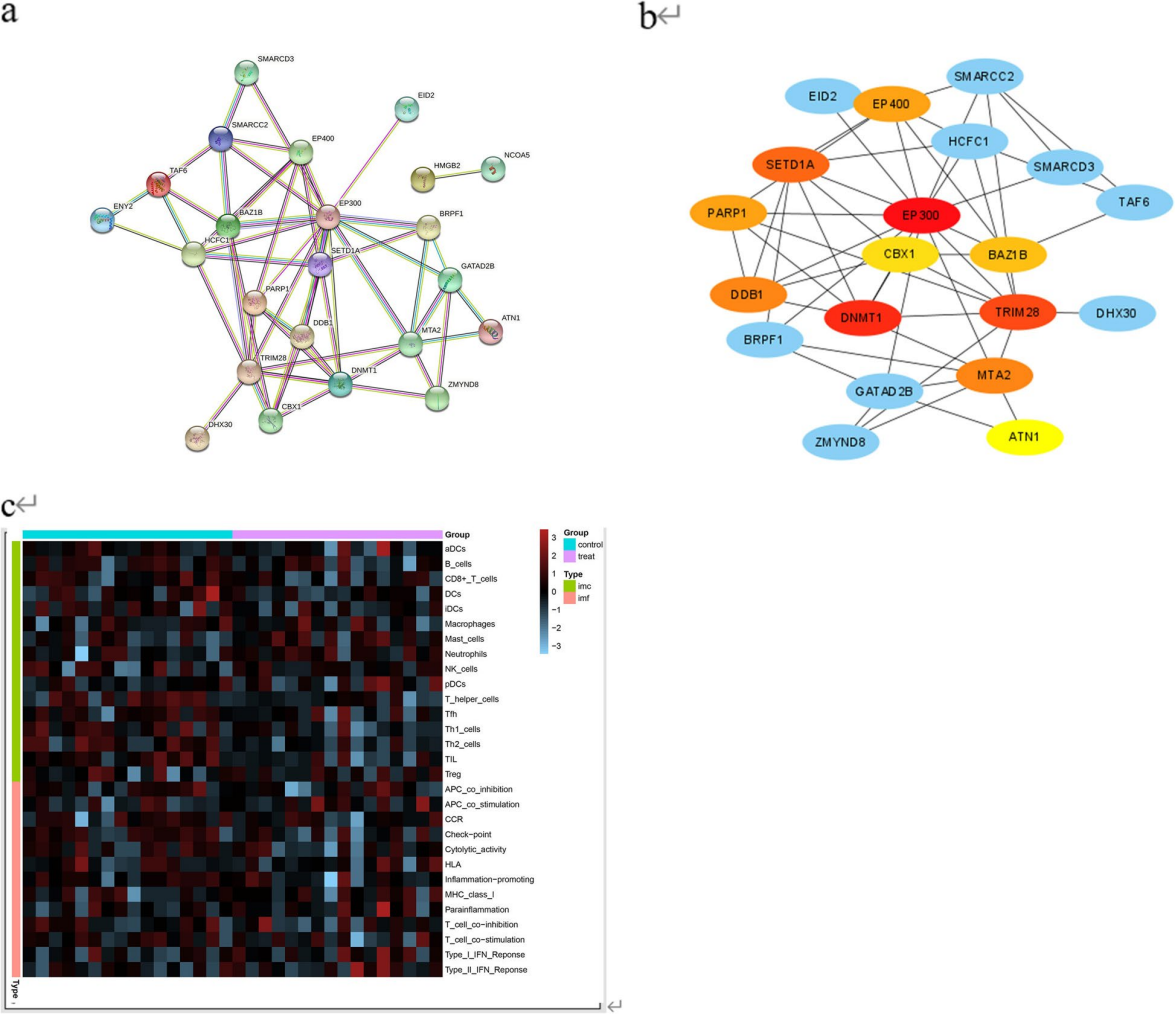
**a** The PPI network of DEGs (**b**) Selection of the first 10 genes with the strongest correlation with AS in chromatin regulator (**c**) Heat map of expression of immune cells and immune function in control and treat groups

In order to investigate the interactions between the differentially expressed genes (DEGs), a protein–protein interaction (PPI) network was constructed using Cytoscape software (Fig. 2a). The most significant module of this network was identified and visualized in Fig. 2b. Using the Network Analyzer plugin, we identified 10 genes as hub genes with degrees ≥ 10, indicating that they play important roles in regulating gene expression in the context of ankylosing spondylitis (AS). These hub genes were further analyzed and their names, abbreviations, and functions are provided in Table 2.

**Table 2:** | Functional roles of 10 hub genes with degree ≥ 10

No	Gene symbol	Full name	Function
1	EP300	E1A Binding Protein P300	Functions as histone acetyltransferase and regulates transcription via chromatin remodeling
2	DNMT1	Gene—DNA Methyltransferase 1	Methylates CpG residues. Preferentially methylates hemimethylated DNA. Associates with DNA replication sites in S phase maintaining the methylation pattern in the newly synthesized strand
3	TRIM28	Tripartite Motif Containing 28	Mediates gene silencing by recruiting CHD3, a subunit of the nucleosome remodeling and deacetylation (NuRD) complex, and SETDB1 (which specifically methylates histone H3 at 'Lys-9' (H3K9me)to the promoter regions of KRAB target genes
4	SETD1A	SET Domain Containing 1A, Histone Lysine Methyltransferase	Part of chromatin remodeling machinery, forms H3K4me1, H3K4me2 and H3K4me3 methylation marks at active chromatin sites where transcription and DNA repair take place
5	DDB1	Damage Specific DNA Binding Protein 1	Protein, which is both involved in DNA repair and protein ubiquitination, as part of the UV-DDB complex and DCX (DDB1-CUL4-X-box) complexes
6	MTA2	Metastasis Associated 1 Family Member 2	May be involved in the regulation of gene expression as repressor and activator. The repression might be related to covalent modification of histone proteins
7	EP400	E1A Binding Protein P400	Component of the NuA4 histone acetyltransferase complex which is involved in transcriptional activation of select genes principally by acetylation of nucleosomal histones H4 and H2A
8	PARP1	Gene—Poly(ADP-Ribose) Polymerase 1	Poly-ADP-ribosyltransferase that mediates poly-ADP-ribosylation of proteins and plays a key role in DNA repair
9	BAZ1B	Bromodomain Adjacent To Zinc Finger Domain 1B Chromobox 1	Atypical tyrosine-protein kinase that plays a central role in chro- matin remodeling and acts as a transcription regulator
10	CBX1	Chro- mobox 1	Recognizes and binds histone H3 tails methylated at ’Lys-9’, lead- ing to epigenetic repression. Interaction with lamin B receptor (LBR) can contribute to the association of the heterochromatin with the inner nuclear membrane.

### Analysis of immune cells and immune function includes differential analysis and correlation analysis of hub genes

**Fig. 3:**
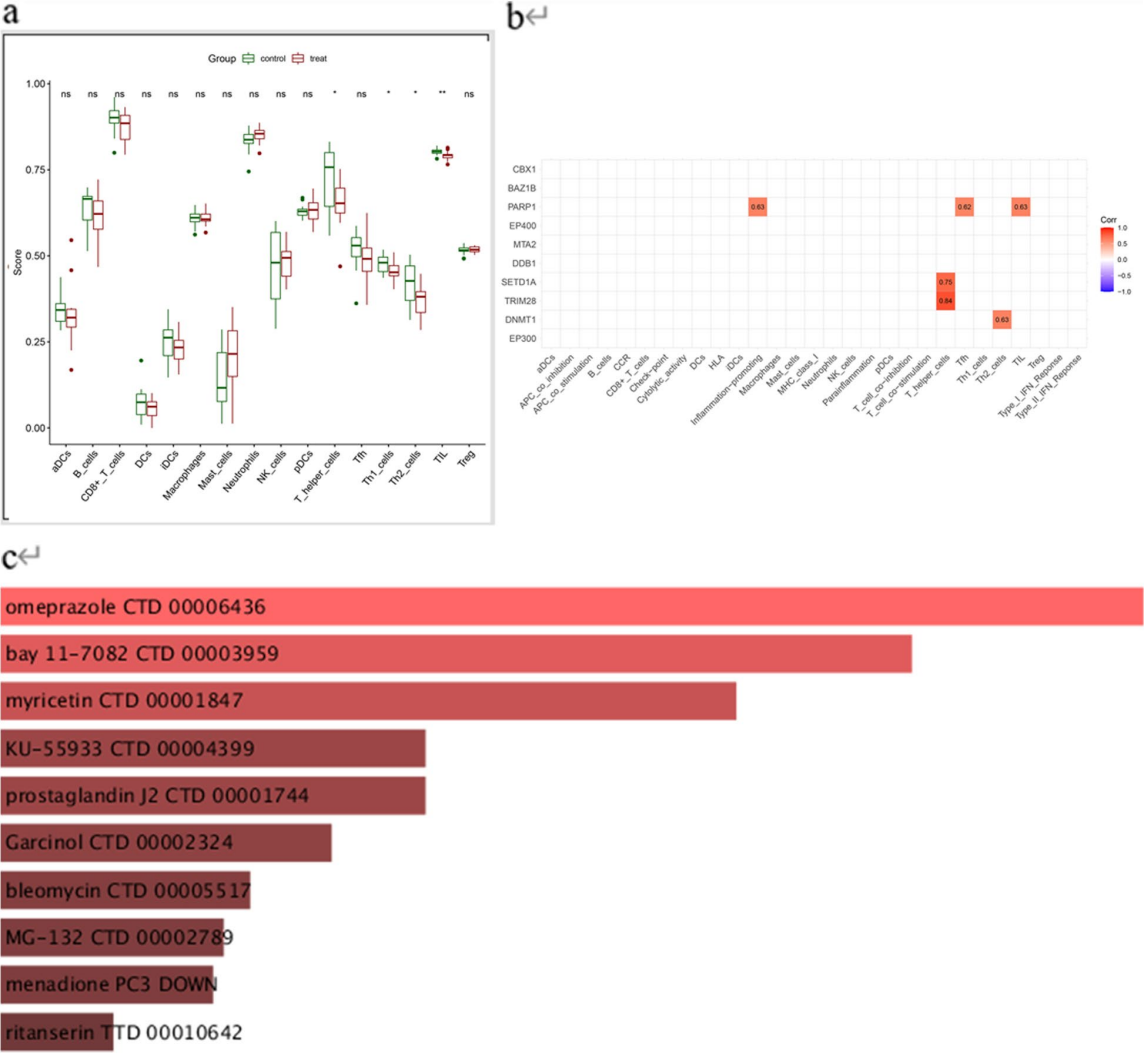
**a** Differential expression of immune cells in the control and treat groups (**b**) Analysis of the correlation between hub genes and immunity (**c**) Ten drugs predicted to treat AS

We matched and scored the AS dataset with immune-related genes. Plot the results as a heat map to show(Fig. 2c). Differential analysis of the expression of genes related to immune cells was done in the experimental and control groups of AS, reflecting the factors in immunity related to the development of AS(Fig. 3a). There were significant differences between experimental and control groups in the gene expression of immune cells regarding T helper cells, Th1_cells, Th2_cells, and TIL(Tumor-infiltrating lymphocytes) expression (*P* < 0.05). No significant differences in gene expression for immune function. The results of hub gene and immune correlation analysis showed a close association with Inflammation-promoting, T helper cells, Tfh(Follicular B helper T cells), Th2 cells, and TIL (Fig. 3b).

### Predicting 10 drugs associated with the hub gene

To identify potential drug treatments for AS, we utilized the hub genes that were previously screened. Through drug prediction analysis, we identified the top ten drugs that were closely associated with the pivotal genes. These drugs were then selected as potential therapeutic options for the treatment of AS. The results are displayed in Fig. 3c, providing valuable information for future drug development and clinical research in the field of AS treatment.

## Discussion

### Current research

Ankylosing spondylitis is a chronic inflammatory disease that primarily affects the spine and sacroiliac joints [11]. Its pathogenesis involves various factors, including genetic, immune, and environmental factors. AS is undoubtedly one of the most heritable illnesses, as indicated by high monozygotic twin consistency (63%) and familial aggregation studies, which suggest a heritability of over 90% [12]. Although HLA-B27 plays an undisputed key role in the disease's pathogenesis, recent estimates suggest that it accounts for only 20–25% of total heritability and 40% of the genetic risk. In the general population, less than 5% of HLA-B27 carriers develop the disease [3]. However, no better diagnosis or treatment is currently available for AS, placing severe existential and economic stress on patients and society.

### Molecular mechanisms of chromatin regulators and immune direction

In this study, we investigated the molecular mechanisms of AS by exploring chromatin regulators and immune direction in order to find new therapeutic ideas. To achieve this goal, we extracted chromatin regulators-related genes from the dataset and screened differentially expressed genes (DEGs) using logFC|filter = 0.2, adjusted to *p* < 0.05. We identified five significantly up-regulated genes and 24 significantly down-regulated genes. To further understand the molecular mechanism of AS, we performed GO enrichment analysis and found that the DEGs were mainly related to histone modification, chromatin organization, transcription coregulator activity, transcription coactivator activity, histone acetyltransferase complex, and protein acetyltransferase complex. The nucleosome is the basic unit of chromatin, consisting of DNA wrapped around a histone octamer (two copies of H2A, H2B, H3, and H4). The histone tails protruding from the nucleosomes can be affected by the addition of various chemical groups, also known as histone marks [13]. These modifications, including methylation, acetylation, phosphorylation, and ubiquitination, can act as synergistic, complementary, or antagonistic signals that dynamically regulate the intracellular transcriptional outcome by altering chromatin structure and regulating the accessibility of DNA to transcription factors [14]. Histone marks allow ab initio prediction of non-coding regulatory sequences, such as enhancers, which are key elements involved in cell-specific transcription regulation through long-distance interactions with promoter sequences [15]. Malfunctioning enhancers are involved in many genetic and developmental disorders due to abnormal gene expression. DNA methylation mostly involves AS-associated SNPs whose location correlates with enhancer features. Farh et al. integrated candidate causal variant prediction based on intensive genotyping data and enhancer mapping of different immune cell types using H3K27ac distribution and detected an enrichment of relevant SNPs in active enhancers of Th0, Th1, and Th17 cells [16]. Histone modifications are associated with many diseases because they affect the expression levels of many genes simultaneously [17]. The overall level of acetylated histones in cells depends on the balance between histone acetyltransferase (HAT) and histone deacetylase (HDAC). Changes in HAT/HDAC activity were detected in AS patients treated with TNFα inhibitors [18]. Increased levels of HDAC3, which regulates the c-Jun N-terminal kinase pathway and NF-κB activity, have also been detected in peripheral blood mononuclear cells from AS patients [19]. These findings suggest that changes in HAT/HDAC activity could serve as a potential diagnostic indicator of disease progression in AS.

Based on the STRING database, we analyzed the PPI network and obtained the top 10 hub genes, including EP300, DNMT1, TRIM28, SETD1A, DDB1, MTA2, EP400, PARP1, BAZ1B, and CBX1. EP300 encodes the adenovirus E1A-associated cellular p300 transcriptional co-activator protein, which functions as a histone acetyltransferase regulating transcription via chromatin remodeling and is important in cell proliferation and differentiation. During macrophage transcriptional activation and differentiation, EP300-catalyzed histone acetylation is followed by the loss of a portion of bound nucleosomes in an SWI/SNF-dependent manner, leading to enhanced gene transcription. In this process, the promoters of PARP1 genes encode proteins involved in DNA repair mechanisms and contribute to the maintenance of genomic stability [20]. Macrophages participate in the pathogenesis of ankylosing spondylitis by producing inflammatory cytokines [21]. DNMT1 encodes an enzyme that transfers methyl groups to cytosine nucleotides of genomic DNA, which is responsible for maintaining methylation patterns following DNA replication, establishing and regulating patterns of methylated cytosine residues. Dysregulation of DNMT1 expression by altering the methylation levels of other target genes may contribute to the development of AS [22]. TRIM28 is a co-repressor that regulates transcriptional activity during necrosis. Activated RIPK3 phosphorylates TRIM28 on serine 473, inhibiting its chromatin-binding activity and promoting trans-activation of NF-κB and other transcription factors, leading to elevated cytokine expression and enhancing immunomodulatory processes such as dendritic cell maturation [23]. Studies have demonstrated the role of dendritic cells in triggering inflammatory tissue responses through activation of the IL-17 axis, and dendritic cells may be associated with AS-related new bone formation [24].

Results of differential analysis based on immune cells and immune function indicated significant differences in the expression of immune cell genes between the experimental and control groups concerning T helper cells, Th1 cells, Th2 cells, and TIL expression. T helper cells play an intermediary role in the immune response by proliferating and spreading to activate other types of immune cells that produce direct immune responses. CD4 is the primary surface marker of helper T cells, and mature T h cells express CD4 on their surface and are called CD4 + T cells. Proliferating helper T cells differentiate into effector T cells, which develop into two major cell subtypes: Th1 and Th2 cells. The imbalance of cytokines secreted by Th1 and Th2 cells is critical for the pathogenesis and progression of autoimmune diseases [25]. Th1 cells secrete cytokines such as IFN-g and TNF-a, promoting macrophage-induced resistance to infection and may contribute to autoimmune diseases in certain organs [26]. Th2 cells secrete cytokines such as IL-4 and IL-10, which promote B lymphocyte proliferation, differentiation, antibody production, and participation in humoral immune responses. Abnormal expression of Th2 cytokines has been associated with diseases such as asthma [27]. The body normally balances the amounts of Th1 and Th2, but a state of Th1/Th2 imbalance is found in various autoimmune diseases. When the number of Th1 cells increases, TNF-α is upregulated, which prevents normal bone and cartilage growth and causes chronic inflammation. Elevated TNF-α secretion is thought to play an important role in the pathogenesis of autoimmune diseases, including AS. Therefore, biological treatments for AS often use TNF-α antagonists to alleviate symptoms [28]. Cytokines released by Th1 and Th2 cells can antagonize each other, and the Th1 cytokine IFN-g can antagonize the secretion of the Th2 cytokines IL-4 and IL-10, thus further inhibiting the Th2 response [29]. Serum IL-4 and IL-10 levels were significantly lower in patients with active AS, while TNF-a and IFN-g levels were higher, suggesting that an imbalance in the number of Th1/Th2 cells may be involved in the pathogenesis of AS [30]. Micheliolide was found to alleviate AS by inhibiting the activation of NLRP3 inflammatory vesicles and maintaining the Th1/Th2 balance by regulating the NF-κB signaling pathway [31]. In hub gene and immune correlation analysis, PARP1 was closely associated with inflammation-promoting, Tfh, and TIL. In macrophages, PARP1 regulates transcription, signaling, inflammatory vesicle activity, metabolism, and redox homeostasis, supporting macrophage polarization towards a pro-inflammatory phenotype (M1) and driving host defense against pathogens involved in the pathogenesis of ankylosing spondylitis [32].

### Drug forecasting

Hub gene analysis for drug prediction BAY 11–7082, an NF-κB inhibitor, reduced the expression of PNF-κB, NLRP3, tumour necrosis factor-α (TNF-α), interleukin (IL)-6 and IL-1β, attenuated the phosphorylation of signal transducer and activator of transcription 3 (STAT3) and decreased the level of IL-23 [33]. Myricetin is a plant-derived flavonoid with anti-inflammatory properties [34]. Myricetin has been shown to inhibit TNF-α-induced NF-κB activation in cells, thereby reducing their inflammatory response [35]. The use of BAY 11–7082 and myricetin to reduce the expression of TNF-α and thus alleviate the symptoms of AS is a novel therapeutic idea.

## Conclusions

The study of recently identified genes, EP300, DNMT1, and TRIM28, associated with chromatin regulation may provide new perspectives for understanding AS pathogenesis and serve as biomarkers for AS diagnosis. Additionally, BAY 11–7082 and myricetin provide new drug avenues for AS treatment. This study provides the first analysis of the possible mechanisms underlying AS occurrence from the perspective of chromatin regulator-related genes, as well as an analysis of differential gene expression in immune cells and immune functions. The results of this study not only confirm previous findings but also offer new directions for the diagnosis and treatment of AS. Validation through cell and animal experiments will be conducted in later stages.

## Data Availability

The datasets generated and/or analysed during the current study are available in the [Gene Expression Omnibus (GEO)] repository, [https://www.ncbi.nlm.nih.gov/geo/query/acc.cgi?acc=GSE25101].

## Abbreviations

AS: Ankylosing spondylitis
USA: United States of America
HLA: Human leukocyte antigen
MHC: Major histocompatibility complex
ERAP1: Endoplasmic reticulum aminopeptidase 1
ERAP2: Endoplasmic reticulum aminopeptidase 2
DEGs: Differentially expressed genes
GEO: Gene Expression Omnibus
PPI: Protein–protein interaction
STRING: Search Tool for the Retrieval of Interacting Genes/Proteins
cytoHubba: Cytoscape plugin for identifying network hub genes
TNF-α: Tumor necrosis factor alpha
AY 11–7082: A synthetic inhibitor of NF-kB activation
NF-kB: Nuclear factor kappa-light-chain-enhancer of activated B cells
BP: Biological processes
MF: Molecular function
CC: Cell components
Th1 cells: T helper 1 cells
Th2 cells: T helper 2 cells
TIL: Tumor-infiltrating lymphocytes
P: Probability value
HLA-B27: Human leukocyte antigen-B27
GO: Gene ontology
NSAIDs: Nonsteroidal anti-inflammatory drugs
DMARDs: Disease-modifying antirheumatic drugs
H2A: Histone H2A
H2B: Histone H2B
H3: Histone H3
H4: Histone H4
SNPs: Single nucleotide polymorphisms
Th0: T-helper cell 0
Th17: T-helper cell 17
HAT: Histone acetyltransferase
HDAC: Histone deacetylase
EP300: E1A Binding Protein P300
DNMT1: DNA methyltransferase 1
SWI/SNF: Switch/sucrose non-fermentable
PARP1: Poly(ADP-ribose) polymerase 1
IFN-g: Interferon-gamma
IL: Interleukin
NLRP3: NOD-like receptor family pyrin domain containing 3
STAT3: Signal transducer and activator of transcription 3
Tfh: T follicular helper
TIL: Tumor-infiltrating lymphocyte
